# Longitudinal analysis of anti-SARS-CoV-2 S-RBD IgG antibodies before and after the third dose of the BNT162b2 vaccine

**DOI:** 10.1038/s41598-022-12750-z

**Published:** 2022-05-23

**Authors:** Bruna Lo Sasso, Luisa Agnello, Rosaria Vincenza Giglio, Caterina Maria Gambino, Anna Maria Ciaccio, Matteo Vidali, Marcello Ciaccio

**Affiliations:** 1 Department of Biomedicine, Neurosciences and Advanced Diagnostics, Institute of Clinical Biochemistry, Clinical Molecular Medicine and Clinical Laboratory Medicine, University of Palermo, 90127 Palermo, Italy; 2 Department of Laboratory Medicine, Azienda Ospedaliera Universitaria Policlinico “P. Giaccone”, 90127 Palermo, Italy; 3 Unit of Clinical Biochemistry, University of Palermo, 90127 Palermo, Italy; 4 Foundation IRCCS Ca’ Granda Ospedale Maggiore Policlinico, 20122 Milan, Italy

**Keywords:** Immunology, Infection, Infectious diseases

## Abstract

Immunosurveillance by evaluating anti-spike protein receptor-binding domain (S-RBD) antibodies represents a useful tool to estimate the long immunity against Severe Acute Respiratory Syndrome CoronaVirus 2 (SARS-CoV-2) infection. The aim of this study was to evaluate the kinetics of antibody response in vaccine recipients. We measured anti-S-RBD IgG levels by indirect chemiluminescence immunoassay on Maglumi 800 (SNIBE, California) in 1013 healthy individuals naïve to SARS-CoV2 infection after two and three COVID-19 vaccine doses. We found that anti-S-RBD IgG levels are higher in females than males. Antibody levels gradually decrease to a steady state after four months since the peak, and the decay is independent of age, sex, vaccine doses, and baseline antibodies titer. The third dose induces a high anti-S-RBD IgG reactivity in individuals with previous high responses and triggers a moderate-high anti-S-RBD IgG reactivity. The assessment of anti-S-RBD IgG levels is essential for monitoring long-term antibody response. A third SARS-CoV-2 vaccine dose is associated with a significant immunological response. Thus, our results support the efficacy of the vaccine programs and the usefulness of the third dose.

## Introduction

The pandemic is still ongoing two years after the first severe acute respiratory syndrome coronavirus 2 (SARS-CoV-2) outbreak. Since then, many advances in both the understanding of pathophysiological mechanisms and the disease course have been made, but many questions are unsolved, such as the potential long-term sequelae^1–5^.

The introduction of vaccines against SARS-CoV-2 has changed the course of the pandemic worldwide by reducing both viral transmission and disease burden. It should be mentioned that vaccines can limit but not prevent the contraction of the SARS-CoV-2 infection. Noteworthy, vaccines provide strong protection against severe coronavirus disease (COVID-19), COVID-19-related hospitalizations, and mortality, as shown by the observational study performed in Israel using national surveillance data^6^. Indeed, most COVID-19 vaccine recipients contracting SARS-CoV-2 infection are completely asymptomatic or develop mild symptoms, such as cold and fever. Thus, the characteristics of SARS-CoV-2 infection strongly differ between vaccinated and unvaccinated individuals^7^. Overall, the efficacy of the COVID-19 vaccine is unquestionable, but the long-term antibody response over time remains an open question.

The efficacy of a vaccine can be assessed by different methods, including the evaluation of humoral response through the measurement of circulating antibody levels. Antibody titers represent a reliable immunological correlate of protection (CoP) for assessing the individual level of protection against infection^8^. Natural SARS-CoV-2 infection and vaccination induce a robust humoral and cellular immune response. The activated B-cells produce antibodies against different antigens and epitopes of SARS-CoV-2, mainly nucleocapsid (N) protein, spike (S) protein, or the receptor-binding domain (RBD) of S. The latter has a key role in SARS-CoV-2 infection because it is expressed on the virion surface and mediates virus entry into target cells through the interaction with the receptor angiotensin-converting enzyme 2 (ACE2). Thus, IgG antibodies recognizing the RBD of the S protein (anti-S-RBD) have neutralizing functions. Wu et al. showed that the anti-S-RBD IgG titers significantly correlate with neutralizing activity and are associated with early virus control, highlighting the relevance of such antibodies as a CoP^9^.

Thus, evaluating anti-RBD-S IgG titers provides precious information on individual immunity against SARS-CoV-2 infection. It is well known that both infection and vaccine induce the production of anti-S-RBD IgG, with most patients becoming seropositive within 15–21 days and then progressively decaying to a steady-state^10,11^. The decay in circulating antibodies has raised questions concerning the necessity to improve the protection against SARS-CoV-2 infection by administering a third dose of vaccine, also named the booster dose. Israel was the first country worldwide to approve the administration of the booster dose since July 2021. Then, other countries, including Italy, have joined this initiative. To date, the effectiveness of the booster dose has yet to be poorly assessed. Understanding the protection gained by a booster dose is critical for guiding vaccine strategies, with a significant impact on public health policy to mitigate the pandemic. It is essential to implement an effective vaccine program and understand how long immunity against SARS-CoV-2 persists in infected individuals, in vaccinated healthy individuals, and whether the antibodies produced in the two categories provide protective immunity against SARS-CoV-2 and its variants.

This study aimed to assess the kinetic of anti-S-RBD IgG antibody levels in vaccinated individuals after two and third BNT162b2 vaccine doses.

## Material and methods

### Study population

This is an observational, single-center study performed at the University Hospital “P. Giaccone” of Palermo, Italy. All consecutive individuals presenting to the Laboratory Medicine Unit to measure anti-S-RBD IgG levels, from January to November 2021, with at least two measurements, were enrolled in the study.

The study cohort included 1013 healthy individuals naïve to SARS-CoV-2 infection. All individuals received two doses of the BNT162b2 vaccine (Pfizer-BioNTech) twenty-one days apart. A sub-group of individuals also received the third dose of the BNT162b2 vaccine.

The study was conducted according to the guidelines of the Declaration of Helsinki and approved by the Institutional Review Board of the University Hospital of Palermo (nr 10, 25 November 2020). Informed consent was obtained from all individuals involved in the study.

### Anti S-RBD IgG measurement

The serum anti-S-RBD IgG levels were measured on fresh samples obtained after centrifugation for 15 min at 4000xg at room temperature of whole blood collected in dry tubes. The measurement was performed by indirect chemiluminescence immunoassay on Maglumi 800 (SNIBE-Shenzhen New Industries Biomedical Engineering Co., Ltd, Shenzhen, China) instrumentation, according to the manufacturer’s instructions. The assay has a limit of detection of 0.7794 Binding Antibodies Units (BAU)/mL, as declared by the manufacturer. The unit of measurement used is in accordance with the latest notification received from World Health Organization (WHO) (Notice WHO Standard (20/136) Unit Conversion-RN21040201).

### Statistical analysis

Statistical analyses were performed by R Language v.4.0.3 (R Foundation for Statistical Computing, Vienna, Austria). Quantitative variables were expressed by the median and interquartile range (IQR), while categorical variables by absolute and relative frequency. Differences between groups for continuous variables were estimated respectively by nonparametric Mann–Whitney U-test. The correlation was evaluated by the non-parametric Spearman test.

## Results

A total of 1013 (M:453, F:560) naïve infection individuals underwent multiple anti-S-RBD IgG level measurements post-vaccination. The median age (IQR) was 52 (38–60) years. The median (IQR) anti-S-RBD IgG levels at the first measurement (baseline) were 1206 (522–2601) BAU/mL. Females displayed significantly higher median baseline anti-S-RBD IgG levels than males (1407 vs 1091 BAU/mL, *p* = 0.003). No association was found between age and baseline anti-S-RBD IgG levels.

**Figure 1:**
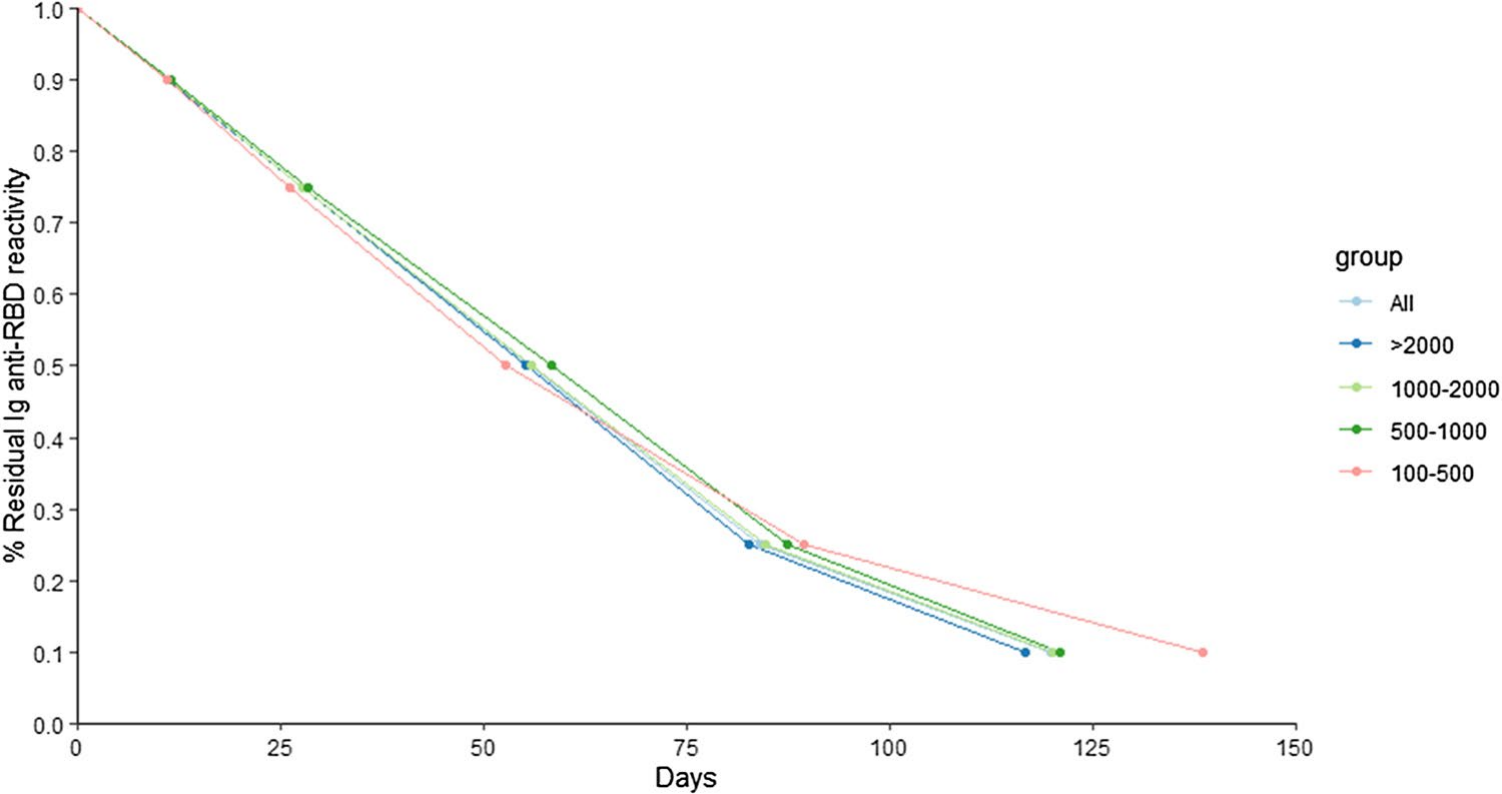
Line plot for the % residual IgG anti-S-RBD reactivity. Each point represents a median. Individuals were sub-grouped according to their baseline anti-S-RBD IgG reactivity.

Out of these 1013 individuals, 550 (54%) had two anti-S-RBD IgG measurements, 377 (37%) three-four and 86 (9%) more than four measurements. Decreasing kinetic was evaluated considering different baseline anti-S-RBD IgG levels, respectively > 2000 BAU/mL, 1000–2000 BAU/mL, 500–1000 BAU/mL and 100–500 BAU/mL (Table 1). A similar trend was observed when kinetic was evaluated considering all anti-S-RBD IgG baseline levels or when individuals were subgrouped on the basis of the baseline values (Table 1). Kinetic curve overlapping is clearly visible in Fig. 1. From Table 1 and Fig. 1, only a minor difference in time to reach 10% residual reactivity for group 100–500 BAU/mL was observed, likely due to the reduced number of individuals used for the calculation (191–155 = 36 individuals). Interestingly, no substantial difference was observed in kinetics between males and females; only a small difference in the number of days to reach 10% residual reactivity was evident, respectively (considering all baseline IgG levels, 90% M:11.0 vs F:11.2, 75% M:27.5 vs F:27.9, 50% M:55.3 vs F:55.7, 25% M:84.0 vs F:84.2, 10% M:114.4 F:126.2).

**Table 1:** % Residual IgG anti-S-RBD reactivity. Each cell reports the numbers of individuals considered for the analysis, the number of individuals excluded from the analysis since the reactivity at the last timepoint was still higher and the descriptive statistics expressed as median and interquartile (IQR).

%Residual IgG anti-S-RBD reactivity					
Baseline					
Anti-S-RBD IgG BAU/mL	90%	75%	50%	25%	10%
**All individuals**					
*N* considered	1013	1013	1013	1013	1013
*N* excluded	19	39	99	257	515
Days	11.1 (9.5–14.6)	27.7 (23.6–35.4)	55.6 (47.6–68.9)	84.1 (74.4–104.1)	119.9 (97.5–153.1)
> 2000					
N considered	342	342	342	342	342
*N* excluded	2	4	9	40	117
Days	11.1 (9.5–14.0)	27.7 (23.8–34.8)	55.1 (47.6–68.2)	82.6 (71.8–100.6)	116.7 (95.1–149.4)
**1000–2000**					
*N* considered	232	232	232	232	232
*N* excluded	2	5	7	26	101
Days	11.2 (9.9–13.0)	27.8 (24.5–32.3)	55.8 (49.9–64.6)	84.7 (76.4–97.5)	120.1 (99.9–145.9)
**500–1000**					
*N* considered	194	194	194	194	194
*N* excluded	2	2	8	44	89
Days	11.4 (9.9–14.5)	28.5 (24.7–36.4)	58.3 (49.7–72.5)	87.5 (76.2–110.7)	121.1 (97.0–157.7)
**100–500**					
*N* considered	191	191	191	191	191
*N* excluded	8	18	44	101	155
Days	11.0 (7.9–16.2)	26.2 (18.9–37.4)	52.7 (36.6–71.0)	89.4 (71.5–112.1)	138.5 (112.7–165.6)

**Figure 2:**
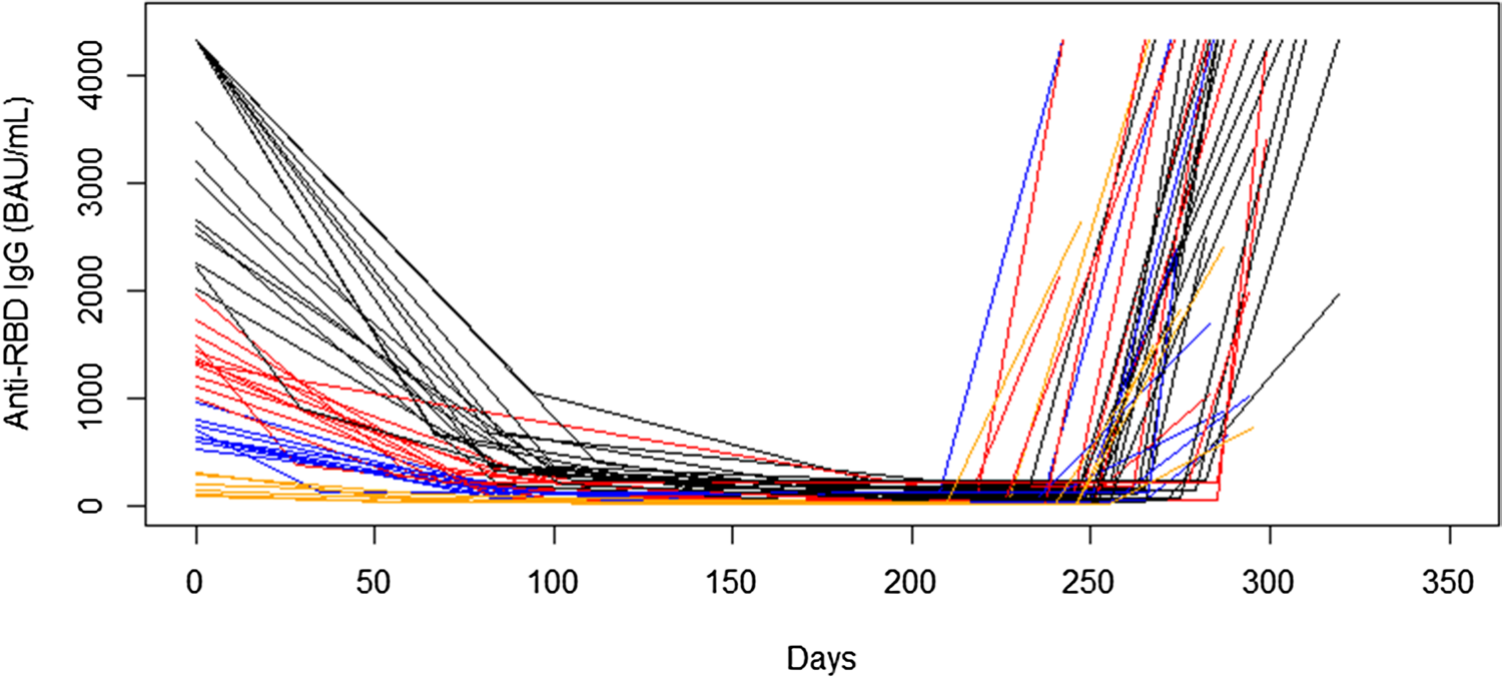
Line plot for the IgG anti-S-RBD reactivity kinetics before and after the booster dose. Days were counted from the first measurement (about 1 month after immunization). For each line, the point at the beginning of the last ascending segment represents the day of the booster dose. The colour of the line represents anti-S-RBD IgG levels reached after immunization (black: > 2000 BAU/mL, red: 1000–2000 BAU/mL, blue: 500–1000 BAU/mL, orange: < 500 BAU/mL). IgG levels are truncated at 4330 BAU/mL, the maximum measurable level (samples with higher titers were not diluted).

In a minority of individuals (*n* = 48), anti-S-RBD IgG levels were also measured after the booster dose administration. In 44 out of 48 individuals, kinetics data were available with several time points, from February 2021 to October–November 2021, when booster dose was administered, ranging between 2 and 8 (median five time points). The median interval between the third dose and the next IgG measurement was 35 days. In Fig. 2, the anti-S-RBD IgG kinetics before and after the booster dose is reported. Individuals were subgrouped according to their baseline (time 0) anti-S-RBD reactivity, into those displaying > 2000 BAU/mL (*n* = 17), 1000–2000 BAU/mL (*n* = 12), 500–1000 BAU/mL (*n* = 9) and < 500 BAU/mL (*n* = 6). 16 out of 17 individuals with baseline anti-S-RBD reactivity > 2000 BAU/mL after the booster dose still displayed comparable high levels (> 2000 BAU/mL), while 1 individual showed 1970 BAU/mL (Table 2, Fig. 2). 11 out of 12 individuals with baseline anti-S-RBD IgG reactivity of 1000–2000 BAU/mL after the booster dose displayed higher levels than those at baseline, with up to 10 individuals showing a high reactivity > 2000 BAU/mL. A single individual after the booster dose displayed a lower reactivity (1027 vs baseline 1482 BAU/mL) (Table 2, Fig. 2). All 9 individuals with baseline anti-S-RBD IgG reactivity of 500–1000 BAU/mL after the booster dose showed a higher reactivity (> 1000 BAU/mL), with 5 out of 9 displaying anti-S-RBD IgG > 2000 BAU/mL (Table 2, Fig. 2). All 6 individuals with baseline anti-S-RBD IgG reactivity < 500 BAU/mL after the booster dose showed a higher reactivity (> 500 BAU/mL), with 3 out of 6 displaying anti-S-RBD IgG > 2000 BAU/mL (Table 2, Fig. 2).

**Table 2:** Anti-S-RBD IgG reactivity after immunization (or baseline, column 1) and after the booster dose (column 2).

After immunization	After booster dose
> 2000 BAU/mL (*n* = 17)	> 2000 BAU/mL (*n* = 16)
	< 2000 BAU/mL (*n* = 1)
1000–2000 BAU/mL (*n* = 12)	> 2000 BAU/mL (*n* = 10)
	> 1000 BAU/mL (*n* = 11)
	< 1000 BAU/mL (*n* = 1)
500–1000 BAU/mL (*n* = 9)	> 2000 BAU/mL (*n* = 5)
	> 500 BAU/mL (*n* = 9)
	< 500 BAU/mL (*n* = 0)
< 500 BAU/mL (*n* = 6)	> 2000 BAU/mL (*n* = 3)
	> 500 BAU/mL (*n* = 6)
	< 500 BAU/mL (*n* = 0)

Individuals were sub-grouped according to their reactivity after immunization (rows). Columns 2 reports, for each group, the number of patients with high anti-S-RBD reactivity (post-dose IgG > 2000 BAU/mL), comparable reactivity (post-dose anti-S-RBD IgG as baseline) and lower reactivity (post-dose anti-S-RBD IgG < baseline).

It is noteworthy that up to 4 out of 48 individuals investigated did not display an increase in anti-S-RBD IgG reactivity after the booster dose (anti-S-RBD IgG < 25 BAU/mL). In 2 out of 4 individuals, a measurement carried out in February 2021 confirmed a low anti-S-RBD IgG response in these patients. In the two remaining individuals, anti-S-RBD IgG levels close to immunization were not available. We were not able to establish if individuals displayed an anti-S-RBD IgG reactivity before (in these two individuals, only measurements in the period August–September 2021 were available, and we could not discriminate if the low titers observed were due to a no response or to an already diminished response).

## Discussion

Vaccines represent the most effective weapon to control the COVID-19 pandemic, and monitoring their effectiveness is critical to assessing individual protection against SARS-CoV-2 infection. The gold standard for evaluating vaccine efficacy is represented by neutralizing assays, such as the plaque reduction neutralization test (PRNT), which measures neutralizing antibody by in vitro virus neutralization^12^. However, such methods have several disadvantages, including a long turn-around time and the need for bio-safety level 3 containment^13,14^. Thus, they cannot be used routinely in clinical practice. The measurement of circulating antibodies against SARS-CoV-2 by commercially available assays based on enzyme‐linked immunosorbent assay (ELISA) or chemiluminescence immunoassay (CLIA), represents a reliable alternative^15^. Indeed, a robust correlation between antibody titers and vaccine efficacy, with higher titers correlating with higher vaccine efficacy, has been recently described by two independent studies^16,17^. Khoury et al.^16^ and Earle et al.^17^ assessed the relationship between vaccine efficacy and antibody titers by analysing data from published clinical studies of several vaccines against SARS-CoV-2. The Authors both found a significant correlation between vaccine efficacy and vaccine-induced antibody activity. These findings are further supported by animal studies and convalescent cohorts^18,19^. Thus, the evaluation of antibodies titers is a reliable, easy-to-perform, and low-cost tool for assessing vaccine efficacy.

Vaccination elicits the immune response, which induces an early peak antibody response that decreases over time. In this large observational study, we investigated the kinetic of anti-S-RBD IgG antibody levels in a cohort of vaccinated with two and three doses. The main findings of our study can be summarised as follows: (i) females have significantly higher baseline levels of antibodies than males; (ii) antibody levels gradually decrease to a steady state after four months since the peak; (iii) anti-S-RBD IgG decay is independent of age, sex, vaccine doses, and baseline antibodies titer; (iv) the booster dose induces a high anti-S-RBD IgG reactivity in individuals with previous high response and trigger a moderate-high anti-S-RBD reactivity also in individuals with an initial low-moderate anti-S-RBD IgG response. Thus, a third SARS-CoV-2 vaccine dose is associated with a significant immunological response.

Our findings are in accordance with previous studies. Brisotto et al. showed a significant antibody decay independent of age and sex in a cohort of 767 healthcare workers 4 months after two-dose vaccination^20^. Similarly, Stamatopoulou et al., in a cohort of 142 infection-naïve healthcare workers, found that the total mean of anti-SARS-CoV-2 IgG levels decreased significantly four months following the second dose^21^. Also, Malipiero et al.^22^ and Matusali et al.^23^, in health care workers who underwent COVID-19 vaccination, showed a marked decline in anti-RBD-IgG levels at six months. Padoan et al. showed a reduction of 90% in anti-SARS-CoV-2 IgG antibody levels, which was independent of age, gender, and previous infection^24^. In the same way, Ibarrondo et al. found a 90% loss of anti-S-RBD levels in the first 91 days after vaccination with BNT162b2^25^, independently of age. Overall, most literature evidence on healthy individuals describes a significant reduction of antibody levels four-six months after the second COVID-19 vaccine dose. Fragile patients, such as patients with haematological malignancies and haemodialysis, show an overall reduced response to anti-SARS-CoV-2 vaccination than healthy individuals^26,27^. Additionally, some Authors reported that fragile patients have a significantly more substantial decline of anti-SARS-CoV-2 antibody titers within 6 months compared to healthy individuals^28,29^.

Recently, some Authors evaluated the immunological response to the booster dose both in healthy and fragile individuals. Eliakim-Raz et al. showed that a third BNT162b2 dose in adults aged 60 years and older was associated with significantly increased levels of anti-S IgG^30^. Similarly, Gilboa et al. demonstrated a rapid and broad immune response to the third BNT162b2 dose, characterised by a significant increase in SARS-CoV-2 RBD IgG levels^31^. Noteworthy, both studies were performed in Israel on healthy individuals aged > 60 years old. Also, Blain et al. in nursing home residents in the French Occitanie region showed a rapid decay of RBD-IgG levels after the second vaccine dose and a significant increase after the third vaccine dose administration^32^. Our study was performed on the Italian population and included individuals with a median age of 52 (38–60) years. Cucunawangsih et al., in 90 health care workers from Siloam Teaching Hospital, Indonesia, with a median age of 31 years, showed that the administration of the third vaccine dose elicited a pronounced antibody response against SARS-CoV-2 infection^33^. Interestingly, the third dose has been proved to be very effective in vulnerable patients. Ducloux et al.^34^ and Espi et al.^35^ reported that the third dose boosted the humoral response in patients on haemodialysis, especially in the low responder to two-doses. Mair et al. evaluated the immune response after the third dose in a large cohort of patients with haemato-oncological disease, showing a meaningfully strengthened humoral immune response^36^. Such finding is in accordance with other studies performed on smaller cohorts^37–39^. Taken together, literature evidence supports the importance of the booster dose both in healthy and vulnerable individuals. The efficacy of the booster dose demonstrated from a serological point of view is corroborated by a clinical point of view. Indeed, Bar-On et al. were the first to show the efficacy of the booster dose by displaying that among 1.137.804 adults aged 60 years and older who received the third dose in Israel, there was a significant reduction of confirmed SARS-CoV-2 infections and severe illness^40^.

Monitoring anti-S-RBD IgG levels as a correlate of protection is helpful for answering important questions about virus neutralization and immunity against SARS-CoV-2. Understanding the kinetic of anti-S-RBD IgG allows healthcare providers and governing bodies to optimise vaccine programs.

The limitations and strengths of this study should be mentioned. The small sample size for third dose evaluation and lack of cellular immunity testing and neutralizing antibody testing are the main limitations. However, the primary analysis included the timing of the anti-SARS-CoV2 IgG antibodies after vaccine administration. Additionally, the patients enrolled were naïve to SARS-CoV-2, but we cannot exclude that asymptomatic or paucisymptomatic patients were also included in the study. However, this potential contamination should not have substantially impacted our result. Indeed, these patients are expected to display higher anti-S-RBD IgG levels (> 2000 or 1000–2000 BAU/mL), and our analysis has clearly shown comparable kinetics in all subgroups. The main strengths are the real-life world study design and that it is the first study evaluating antibody response after booster dose in a wide range of ages. Ongoing surveillance program is required to assess the continuity of our findings over time.

## Data Availability

The datasets generated and analysed during the current study are not publicly available due to restrictions from our Institution but are available from the corresponding author on reasonable request.
